# Zn(II) Complex of Plant Phenolic Chlorogenic Acid: Antioxidant, Antimicrobial and Structural Studies

**DOI:** 10.3390/ma13173745

**Published:** 2020-08-24

**Authors:** Monika Kalinowska, Justyna Sienkiewicz-Gromiuk, Grzegorz Świderski, Anna Pietryczuk, Adam Cudowski, Włodzimierz Lewandowski

**Affiliations:** 1 Department of Chemistry, Biology and Biotechnology, Institute of Civil Engineering and Energetics, Faculty of Civil Engineering and Environmental Sciences, Bialystok University of Technology, Wiejska 45E Street, 15-351 Bialystok, Poland; g.swiderski@pb.edu.pl (G.Ś.); w-lewando@wp.pl (W.L.); 2 Department of General and Coordination Chemistry and Crystallography, Institute of Chemical Sciences, Faculty of Chemistry, Maria Curie-Sklodowska University, Maria Curie-Sklodowska Sq. 2, 20-031 Lublin, Poland; j.sienkiewicz-gromiuk@poczta.umcs.lublin.pl; 3 Department of Water Ecology, Faculty of Biology, University of Bialystok, Ciolkowskiego 1J Street, 15-245 Bialystok, Poland; annapiet@uwb.edu.pl (A.P.); cudad@uwb.edu.pl (A.C.)

**Keywords:** 5-caffeoylquinic acid, chlorogenic acid, zinc, plant phenolic compounds, oxidative stress

## Abstract

The structure of the Zn(II) complex of 5-caffeoylquinic acid (chlorogenic acid, 5-CQA) and the type of interaction between the Zn(II) cation and the ligand were studied by means of various experimental and theoretical methods, i.e., electronic absorption spectroscopy UV/Vis, infrared spectroscopy FT-IR, elemental, thermogravimetric and density functional theory (DFT) calculations at B3LYP/6-31G(d) level. DPPH (2,2-diphenyl-1-picrylhydrazyl), ABTS (2,2′-azino-bis(3-ethylbenzothiazoline-6-sulfonic acid), FRAP (ferric reducing antioxidant power), CUPRAC (cupric reducing antioxidant power) and trolox oxidation assays were applied in study of the anti-/pro-oxidant properties of Zn(II) 5-CQA and 5-CQA. The antimicrobial activity of these compounds against *Escherichia coli*, *Pseudomonas aeruginosa*, *Bacillus subtilis*, *Staphylococcus aureus*, *Salmonella enteritidis* and *Candida albicans* was tested. An effect of Zn(II) chelation by chlorogenic acid on the anti-/pro-oxidant and antimicrobial activities of the ligand was discussed. Moreover, the mechanism of the antioxidant properties of Zn(II) 5-CQA and 5-CQA were studied on the basis of the theoretical energy descriptors and thermochemical parameters. Zn(II) chlorogenate showed better antioxidant activity than chlorogenic acid and commonly applied natural (L-ascorbic acid) and synthetic antioxidants (butylated hydroxyanisol (BHA) and butylated hydroxytoluene (BHT)). The pro-oxidant activity of Zn(II) 5-CQA was higher than the ligand and increased with the rise of the compound concentration The type of Zn(II) coordination by the chlorogenate ligand strongly affected the antioxidant activity of the complex.

## 1. Introduction

Phenolic compounds play an important role in plant tolerance to toxic metals as well as in prevention and reduction of the biotic and abiotic oxidative stress. The chelation of metal ions by phenolic compounds is widely discussed in the literature as a possible hypothesis for the importance of phenolics in toxic metal tolerance in plants [1,2,3]. This mechanism relies on: (i) the secretion of chelating agents by e.g., roots to prevent metal uptake or (b) production of chelating agents to bind metals in the cell wall, symplast or vacuole [1]. For example, an increase in the synthesis of phenolic compounds and activity of shikimate dehydrogenase (SKDH), cinnamyl alcohol dehydrogenase (CAD) and polyphenol oxidase (PPO) was observed in roots and leaves of *Kandelia obovata* under Zn and Cd stress [4]. According to authors the high level of Zn and Cd activated the phenolic metabolism pathways which participated in heavy metal tolerance process. Moreover the presence of Zn decreased the oxidative stress caused by Cd. Chlorogenic acid, among other phenolics, was found in the roots in woody plants as potential aluminum-detoxifying agents [5]. The study of Mongkhonsin et al. revealed that not only the increase in the level of phenolic compounds but also the lignification process played crucial roles in protecting *G. pseudochina* against an excess of Zn [6]. Among the phenolic compounds that were engaged in the esterification of the cell wall were derivatives of caffeic acid, including chlorogenic acid. The application of bulk X-ray absorption near edge structure (XANES) spectra indicated that Zn cations were bonded by O-ligands which could be phenolic compounds. Studies of other authors also confirmed that the exposure of the plant to toxic metals caused an increase in the level of phenolics which intermediated in lignin biosynthesis [7].

Chlorogenic acid (5-caffeoylquinic acid, 5-CQA) is a phenolic compound found in all part of many plants (seeds, roots, tubers, fruits, leaves, flowers or bark) [8,9,10,11,12,13]. 5-CQA plays an important role in defense against biotic and abiotic stress in plants, and is considered to be an intermediate molecule in lignin biosynthesis pathway [14]. Many studies reported accumulation of chlorogenic acid in tissue exposed to different stress factors, e.g., an increase in salinity [15], bacterial infection [16], ultraviolet (UV-B) radiation [17], toxic metals [18,19] and other related with an increase in production of reactive oxygen species (ROS) which damage cellular membrane and react with plant cell components causing disruption of metabolic pathways.

The metal chelating ability, participation in lignification process and scavenging of free radical are the main important mechanisms of antioxidant activity of 5-CQA involved in the decrease in the ROS level in plants under stress condition. However, the complexation with metal ions may change the antioxidant potential of chlorogenic acid. The alkali metal salts of chlorogenic acid possessed higher antioxidant activity than the ligand alone [10]. The complexation of chlorogenic acid with oxidovanadium(IV) also improved the antioxidant and anti-cancer properties against the human breast cancer cell line SKBR3 [20]. The higher antioxidant activity of metal complexes compared to free phenolic compounds was observed in other cases as well, e.g., Fe(II), Cu(II), Ce(IV), La(III) and oxovanadium(IV) complexes of chrysin; complexes of apigenin with La(III), Mg(II) and luteolin with oxovanadium [21] or calcium complex of gentisic acid [22]. It should be kept in mind that the antioxidants may act as pro-oxidants depending on their concentration or the presence of some metal cations. This may be due to the stabilization of the phenoxyl radicals by metal cations and prolonging their lifetime [23]. The phenoxyl radicals may accelerate lipid peroxidation, disrupt mitochondrial membrane potential or induce DNA damage. In part, this mechanism may also explain the toxic effect of some metals on plants. Therefore the question arises: do metal ions affect the anti-/pro-oxidant properties of chlorogenic acid in plant tissue? What is the stability and structure of formed metal complexes? Do the type of metal-ligand bonding and the structure of metal complexes affect the antioxidant activity of the molecules? The answers will help us to understand the features that make the phenolic compounds’ effective antioxidants and detoxifying agents. It will help to design plants which are resistant to stressful conditions and oxidative stress.

Zinc is as a plant nutrient but at higher concentrations, it shows high toxicity, causes growth inhibition, and affects plant metabolism [24]. Therefore, chelation of zinc by chlorogenic acid plays an important role in protection against oxidative stress and enhances the tolerance to toxic metal stress in plants. Moreover, plant phenolic compounds and their metal complexes that possess strong biological activities are intensively studied as potential new effective antioxidants or antimicrobial agents with low toxicity. There are many studies aiming at searching for plants rich in these compounds and new non-destructive methods for chlorogenic acid extraction [25]. 5-CQA isolated from plant product possesses not only strong antioxidant activity, but among others antimicrobial, anti-inflammatory and anti-cancer [25]. Although the complexing abilities of chlorogenic acid toward different metal cations in aqueous solution and solid state were studied [10,20,26,27,28,29,30], the biological properties of metal chlorogenates (including Zn chlorogenate) are scarcely described. Therefore, the studies of the structure and anti-/pro-oxidant activity of zinc complex with chlorogenic acid are of great importance. The aim of the paper was to synthesize the Zn(II) chlorogenate in the solid state and study the structure and type of interactions between the Zn(II) cation and chlorogenate ligand by the use of UV/Vis, FT-IR, elemental, thermogravimetric analysis and DFT (density functional theory) calculations in Gaussian program. Anti-/pro-oxidant and microbiological activity of Zn(II) chlorogenate was studied and compared with ligand properties. Moreover, the mechanism of antioxidant activity of the complex and the effect of the coordination mode on the antioxidant activity of the complex were studied on the basis of the calculated energy descriptors and thermochemical parameters.

## 2. Materials and Methods

### 2.1. Materials

All chemicals were purchased from Sigma-Aldrich Co. (St. Louis, MO, USA) and used without purification. Only CH_3_OH was bought from Merck (Darmstadt, Germany). *Escherichia coli* (PCM 2857), *Pseudomonas aeruginosa* (PCM 2720)*, Bacillus subtilis* (PCM 2850), *Staphylococcus aureus* (PCM 2267), *Salmonella enteritidis* (NCTC 4776) and *Candida albicans* (PCM 2566-FY) were bought from the Polish Collection of Microorganisms (Wroclaw, Poland) or American Type Culture Collection.

### 2.2. Sample Preparation

The Zn (II) complex of chlorogenic acid was obtained as follows. First, sodium salt of chlorogenic acid was synthesized in a solid state: the aqueous solutions of NaOH (0.05 M) and chlorogenic acid (0.05 M) were mixed in the stoichiometric molar ratio 1:1. The solution was allowed to evaporate on a water bath [10]. The composition of the dry residue was examined by elemental and thermogravimetric analysis. The formula of sodium chlorogenate was C_16_H_17_O_9_Na·1.5H_2_O (results of elemental analysis: exp.%C = 47.84, calc.%C = 47.65; exp.%H = 4.94, calc.%H = 5.00). Then, aqueous solution of the sodium salt at the concentration of 0.05 M was prepared and mixed with aqueous solution of ZnCl_2_ (0.05 M) in the stoichiometric molar ratio 2:1. The nude precipitate occurred immediately. It was filtered and washed with the distilled water. The solid residue was dried in room temperature for 5 days. The results of the elemental analysis showed the formula of the complex to be: Zn(C_16_H_17_O_9_)_2_∙3H_2_O (%C_exp._ = 46.13; %C_calc_ = 46.49; %H_exp._ = 4.78, %H_calc._ = 4.84, %Zn_exp._ = 7.59; %Zn_calc._ = 7.90).

### 2.3. Anti-/Pro-Oxidant Study

The antioxidant activity of the tested compounds was measured by the use of DPPH, ABTS, FRAP and CUPRAC tests. The DPPH assay was described by [31] and our previous work [10]. The solutions of DPPH (C = 60 μM) and tested substances (C = 50 and 500 μM) were prepared in methanol. To the glass tubes the appropriate volumes of tested substances were added, then diluted with methanol and 2 mL of DPPH. The final concentrations of antioxidants were in the range 0.1–70 μM. Each tube was vortexed and incubated in dark place for 1 h at 23 °C. Then the absorbance was measured at 516 nm against methanol (the blank). The control sample was the mixture of 2 mL of DPPH and 1 mL of methanol. The antiradical activity of tested substances was calculated from the equation:

(1) $$\%\;I=\frac{A_{\mathrm{control}\;}-A_{\mathrm{sample}}}{A_{\mathrm{control}}}\times 100\%$$

where: % I—the percent of inhibition of DPPH· radicals, A_control_—the absorbance of the control, A_sample_—the absorbance of the sample. Then, the concentration of the tested substances was plotted versus the % inhibition and the EC_50_ values were read from the curves. The EC_50_ means the concentration of antioxidant that inhibits 50% of the DPPH· radicals.

The aqueous solutions of 2,2′-azino-bis(3-ethylbenzothiazoline-6-sulfonic acid) diammonium salt (ABTS; C = 7 mM) and K_2_S_2_O_8_ (C = 2.45 mM) were mixed in a volumetric ratio 1:1 and left for 16h at 23 °C. The solution of cation radical of ABTS was prepared by mixing 1 mL of the mixture and 60 mL of methanol. 1 mL of methanolic solution of tested substance was added to 1 mL of solution of ABTS·^+^, incubated for 7 min at 23 °C. The final concentration of tested substances was 25 μM. The absorbance was read at λ = 734 nm against methanol. The antiradical activity against ABTS·^+^ was expressed as the percent of ABTS·^+^ inhibition and calculated according to the aforementioned formula.

The FRAP assay determines the ferric-reducing antioxidant activity of antioxidants [10,31]. The FRAP reagents: 0.3 M acetate buffer (pH 3.6), 10 mM 2,4,6-tripyridyl-s-triazine (in 40 mM HCl) and 20 mM FeCl_3_ (in water) were mixed in a volumetric ratio 10:1:1. Then, 3 mL of the mixture was added to 0.4 mL of tested substance (final concentration 50 μM) and incubated for 7 min at 23 °C. The absorbance was measured at 595 nm against blank (i.e., 3 mL of FRAP mixture and 0.4 mL of methanol). The antioxidant activity was expressed as Fe^2+^ equivalents (μM) using the calibration curve prepared for FeSO_4_ (y = 2.0172x − 0.0708; R^2^ = 0.9992).

The CUPRAC assay determines the cupric reducing antioxidant activity of antioxidants. The solutions of CuCl_2_ (C = 10 mM; in water), ammonium acetate (pH = 7; in water) and neocuproine (C = 75 mM; in ethanol) were mixed in a volumetric ration 1:1:1. 3 mL of the mixture was added to 0.5 mL of tested substance and 0.6 mL of distilled water. The final concentration of tested substances was 50 μM. The samples were incubated for 1h at 23 °C. Then the absorbance was read at 450 nm against blank (i.e., 3 mL of CUPRAC mixture, 0.6 mL of water and 0.5 mL of methanol). The antioxidant activity was expressed as trolox equivalents (μM) using the calibration curve prepared for trolox (y = 4.5758x − 0.0271; R^2^ = 0.9919).

The pro-oxidant activity of the tested substances were measured as the rate of oxidation of trolox according to the procedure of Zeraik et al. [32] and described previously [10]. The chemicals were added in the following order: 100 μM trolox, 50 μM H_2_O_2_, 0.01 μM horseradish peroxide in phosphate buffer (pH = 7) and tested substance with a final concentration from 0.025 to 0.15 μM. The mixture was vortexed and incubated at 25 °C. The absorbance measurements (at 272 nm) were made every 5 min through 15 min.

All measurements were taken in five repetition in three independent experiments. The absorbance was measured using an Agilent Carry 5000 spectrophotometer (Agilent, Santa Clara, CA, USA).

### 2.4. Antimicrobial Study

*Escherichia coli, Pseudomonas aeruginosa, Bacillus subtilis, Staphylococcus aureus, Salmonella enteritidis* and *Candida albicans* were grown overnight and then were resuspended in physiological saline to an optical density OD = 0.60 at 600 nm. It corresponds to 5.0 × 10^8^ colony-forming units (CFU)/mL. Microorganisms (0.1 mL of the reconstituted suspension) were seeded onto sterile Mueller-Hinton agar plates. The suitable amounts of the tested compounds (dissolved in an agar medium) were added to give their desired concentration. The range of studied concentration was 0.05–10 mM. The negative controls were agar plates without tested substances. The positive control was gentamycin (in the case of bacteria) or flucanozole (in the case of fungi). The plates were incubated at 37 °C for 24 h. The antimicrobial activity of tested compounds was expressed as MIC (minimum inhibitory concentration).

### 2.5. Statistical Analysis

For parametric data one-way analysis of variance (ANOVA) followed Tukey’s test was applied using Statistica 13.1 program. Results from three independent experiments were expressed as mean ± standard deviation (SD) of mean for parametric data. Significance was considered when *p* ≤0.05.

### 2.6. Structural Studies

The FT-IR spectra for the solid samples were recorded in KBr matrix pellets with an Alfa Bruker spectrometer (Bremen, Germany) within the range of 400–4000 cm^−1^ with the resolution of 2 cm^−1^. UV/Vis spectra of chlorogenic acid and its Zn complex were recorded with UV/VIS/NIR Carry 5000 spectrophotometer. To determine the molar ratio of zinc(II) to 5-CQA the Jobs’ method was used. The spectra were recorded for a solution with different molar ratios of zinc ion and chlorogenic acid, but constant total amount of zinc(II) plus 5-CQA moles. The concentration of 5-CQA was 0.1 mM, the concentration of ZnCl_2_ changed from 0 to 0.09 mM. All solutions were prepared in Tris-HCl buffer (pH = 7.4; C = 50 mM). Thermogravimetric analysis (TG) along with differential scanning calorimetry (DSC) was performed by using Setsys 16/18 thermal analyzer of Setaram Instrumentation brand (SETARAM, Caluire, France). The sample of zinc(II) complex (4.27 mg) was heated in the range of 30–750 °C in ceramic crucible at a heating rate of 10 °C min^−1^ in a flowing air atmosphere (v = 1 dm^3^ h^−1^).

### 2.7. DFT Calculations

The quantum-chemical calculations for Zn chlorogenate were done in B3LYP/6-31G(d) using the GAUSSIAN 09W and GaussView 6 software (Gaussian Inc., Wallingford, CT, USA) package running on a Dell PC computer (Manufacture, Round Rock, TX, USA) [33]. The optimized structure for chlorogenic acid was published before [12]. The geometry, IR, the highest occupied molecular orbital (HOMO) and lowest unoccupied molecular orbital (LUMO), selected chemical reactivity parameters [34] and thermodynamic parameters [35,36] were calculated.

## 3. Results and Discussion

### 3.1. Anti-/Pro-Oxidant Study

The results of reactions of studied compounds with DPPH^•^ radicals were given as the EC_50_ parameter which signifies the concentration of compound that inhibits 50% of the radicals (Table 1). The assay showed that Zn 5-CQA possessed better antioxidant properties (EC_50_ = 5.45 ± 0.37 μM) than the ligand alone (EC_50_ = 7.23 ± 0.76 μM) or natural (L-ascorbic acid EC_50_ = 10.32 ± 0.98 μM) and synthetic antioxidants (i.e., BHA EC_50_ = 13.54 ± 1.61 μM, BHT EC_50_ = 53.14 ± 1.05 μM). Zn 5-CQA was a better scavenger of cation radicals ABTS^•+^ than other studied compounds, including 5-CQA. At the same concentration of studied antioxidants (i.e., 25 μM), Zn 5-CQA inhibited 97.65% of the initial concentration of ABTS^+^, whereas 5-CQA inhibited 89.53%. Two more tests for study the antioxidant activity of the compounds were used, i.e., FRAP and CUPRAC assays, which respectively allowed to determine the ferric and cupric reducing antioxidant activity of the compounds at the concentration of 50 μM. In both tests, Zn 5-CQA showed better reducing properties than 5-CQA and commonly applied antioxidants. The FRAP value for Zn 5-CQA was 385.56 μM Fe^2+^, whereas 216.09 μM Fe^2+^ for 5-CQA and even lower for other antioxidants. The last two antioxidant tests measured the ability of an antioxidant to transfer an electron from the antioxidant to any compound in order to reduce it. The mechanism is called SET (single electron transfer). While the first two assays (DPPH and ABTS) based on mixed mechanism which is commonly described as mechanism involving both SET and HAT (hydrogen atom transfer). The studies of Litwinienko [37,38] and Foti [39,40] revealed that the mechanism of action of antioxidants depended on their chemical structure and the environment and therefore several particular mechanisms were described: (a) SPLET (sequential proton-loss electron-transfer), (b) PCET (proton-coupled electron-transfer), (c) ET-PT (electron-transfer proton-loss) as well as (d) HAT [37,38]. As the polarity of solvent increases, the contribution of the HAT mechanism decreases in favor of the other mechanisms [37,38]. In methanol (DPPH assay) and methanol-aqueous solution (ABTS assay) the studied antioxidants were partially ionized, the phenolate anions were formed, which can rapidly react with radicals, through an electron transfer. In ionizing solvents, slow HAT mechanism was rather marginal [37,38]. Therefore, it could be stated that the studied antioxidants act mainly through electron transfer and the ionization potential (IP) could be one of the important parameters defining the antioxidant potential of these compounds. The ionization potential describes the susceptibility of molecule to ionization, and the lower is the IP, the easier is to detach an electron from the antioxidant. The higher antioxidant property of zinc complex of chlorogenic acid compared with chlorogenic acid may be caused by easier electron abstraction from covalently bound 5-CQA to zinc ion than 5-CQA alone. This was discussed later in the work on the basis of spectroscopic study and quantum-chemical calculations.

**Figure 1:**
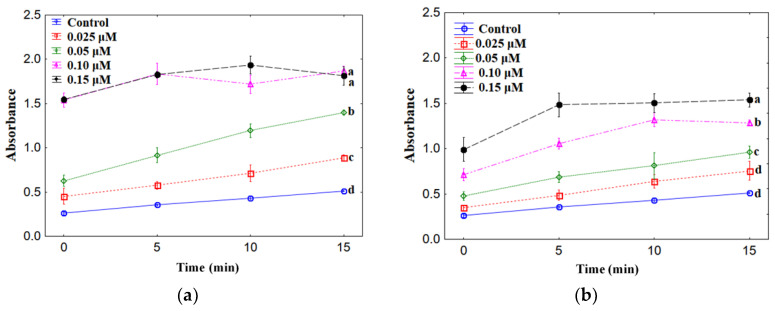
The effect of different concentrations (0.025–0.15 μM) of (**a**) zinc complex of chlorogenic acid and (**b**) chlorogenic acid on the oxidation of trolox. Mean values from three independent experiments ± standard deviation (SD) are shown. The same letter near the means indicates no significant difference (Tukey test, *p* < 0.05).

Zinc chlorogenate was tested for pro-oxidative effect on trolox oxidation in the general procedure for study the pro-oxidant activity of phenolic compounds [32]. The radicals of chlorogenate and chlorogenic acid were produced in their direct reaction with H_2_O_2_ catalysed by the enzyme horse radish peroxide. The formed phenoxyl radicals reacted with trolox which was oxidizing to trolox radicals and then trolox quinones. Whereas the phenoxyl radicals were transformed to phenolic compounds. The results of this study are shown in Figure 1. The rate of trolox oxidation increased with the increase in the concentration of Zn 5-CQA from 0.025 to 0.15 μM. Zn chlorogenate showed higher pro-oxidant activity than chlorogenic acid, especially in the concentration range 0.025–0.15 μM (Figure 1). The maximum of the pro-oxidant activity was reached at higher concentration (in our experiment: 0.10 and 0.15 μM) after maximum 10 min. of the measurement. The mean absorbance of the sample measured after 10 min. reached 1.718 for Zn 5-CQA and 1.321 for 5-CQA, and after 15 min. it was equal 1.936 (Zn 5-CQA) and 1.504 (5-CQA).

### 3.2. Antimicrobial Study

The pro-oxidant activity of chemicals could be an explanation of their antimicrobial property [41,42,43,44]. Therefore, the pro-oxidant activity of phenolic compounds may explain their antimicrobial action in plants as well. The studies revealed that Zn 5-CQA and 5-CQA did not show antimicrobial properties against *E. coli, P. aeruginosa, C. albicans, B. subtilis*, and *S. enteritidis* at the concentration < 10 mM (Table 2). Surprisingly, Zn 5-CQA showed higher activity than 5-CQA against *S. aureus* with the MIC = 1 mM. In the case of the rest of microorganism the same value of MIC was obtained for both 5-CQA and Zn 5-CQA. The antimicrobial properties of chlorogenic acid were widely described. The literature MIC values for 5-CQA differ significantly between each other, i.e., MIC = 14–28 mM against *K. pneumoniae, P. vulgaris, P. aeruginosa, E. faecium, C. albicans,* and *S. cerevisiae* [10], MIC = 23–45 μM for clinically isolated *Stenotrophomonas maltophilia* [45], MIC = 2.2–35.3 mM against *Aeromonas* species isolated from fish [46], MIC = 5.6 mM against *Alicyclobacillus acidoterrestris* [47], MIC = 56 μM in the cases of *S. pneumoniae* and *Shigella dysenteriae*. According to Lou et al. the mechanism of antimicrobial action of chlorogenic acid relies on binding to the outer membrane, disrupting the membrane what causes intracellular potential disorder and release cytoplasm macromolecules, which leads to cell death [25,48]. Generally, 5-CQA shows antimicrobial and anti-biofilm activity through change of the permeability of cell walls of microorganisms [28]. Wang et al. showed that 5-CQA is active toward multi-drug resistant S. aureus (IC_50_ = 96 μM) via inhibition of the activity of sortase A [49]. The formation of metal complexes with ligands of proven antimicrobial activity may enhance their antimicrobial activity. It can be explained by inter alia an increase in the lipophilicity of the complex compared with the ligand. The change of the biological activity of alkali metal salts of chlorogenic acid vs. their lipophilicity was discussed previously [10]. The lipophilicity is an important parameter of antimicrobial agents. Because most of the molecules penetrate the cell membranes by passive diffusion, so they should be lipophilic enough to cross through the biological membranes, but also hydrophilic enough to penetrate the cytoplasm. The increase in the antimicrobial properties of metal complexes of plant phenolic acids compared to ligands itself was described in many papers [21]. E.g. Zn(II) complex of p-coumaric acid possessed higher antimicrobial properties than ligand alone, especially against *S. aureus* (at the concentration of 0.1% in broth culture it caused 77 ± 3% and 75.2% of growth inhibition after 24 and 48 h of incubation, respectively) [50]. In another study, all tested Cu(II), Zn(II), Na(I) complexes of ferulic acid revealed higher antimicrobial activity than ferulic acid [51]. Zn ferulates possessed the strongest antimicrobial properties toward *E. coli, B. subtilis, S. aureus, P. vulgaris* and *C. albicans* (at the concentration of 0.1% in broth culture it caused 90.8–98.9% of growth inhibition).

### 3.3. Structural Studies

#### 3.3.1. FT-IR Spectra

**Figure 2:**
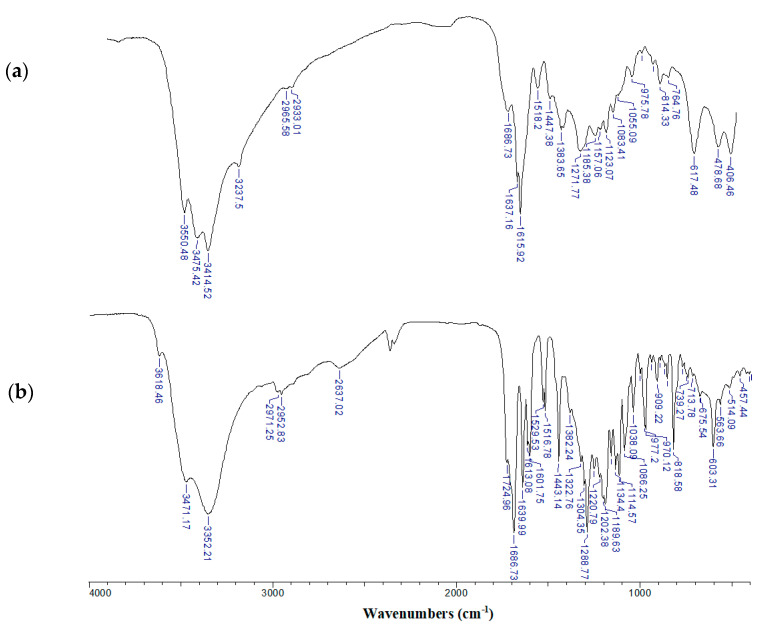
The FT-IR spectra of: (**a**) Zn(II) chlorogenate and (**b**) chlorogenic acid registered in the range of 400–4000 cm^−1^ for solid samples in the KBr matrix pellet.

To characterize the type of interaction between the Zn(II) cation and chlorogenate ligand the FT-IR method was applied. The FT-IR spectra of Zn 5-CQA and 5-CQA were shown in Figure 2, and the assignment of the selected bands were gathered in Table 3. The assignment was based on our previous publications [10,12] concerning chlorogenic acid and alkali metal chlorogenates. The comparison of the FT-IR spectra of synthesized compound and initial ligand gave information about the correctness of the synthesis and the type of metal ion coordination. In the spectrum of 5-CQA there was a strong band at 1725 cm^−1^ (derived from the stretches of the C=O from the carboxylic group) which disappeared in the spectra of Zn complex. Moreover in the spectra of zinc chlorogenate several bands assigned to the vibrations of carboxylate anion appeared, i.e., stretching asymmetric 1616 cm^−1^ and symmetric 1384 cm^−1^, deforming in plane 814 cm^−1^, and out of plane bending at 617 cm^−1^ (Table 3). It confirmed the metal ion coordination through the carboxylate anion. The C=O stretching bands assigned to the ester group were located at the same wavenumbers in the spectra of ligand and zinc complex, what suggested that this group did not participate in metal coordination. The band assigned to the stretching vibrations of the catechol C-O group were significantly shifted to the 1272 cm^−1^ in the spectra of Zn complex, whereas in the spectra of 5-CQA it was located at the 1289 cm^−1^. It may suggest additional metal coordination through the catechol moiety and weakness of the C–O bond strength. The simultaneous metal ion coordination to both carboxylate and catechol moieties and the formation of oligomeric complexes is typical for ligands possessing two coordinating sites. Some other bands present in the spectra of Zn 5-CQA were slightly shifted or disappeared compared with the spectra of 5-CQA. It means that the coordinated metal affects the structure of the quinic and caffeic acid moieties.

#### 3.3.2. Thermogravimetric (TG)/Differential Scanning Calorimetry (DSC) Analysis

The conducted studies revealed high anti-/pro-oxidant properties of Zn 5-CQA what creates the possibility to applicate Zn 5-CQA as antioxidant or pro-oxidant in e.g., plant protection against oxidative stress or in food industry or pharmacy as a preservative or diet supplement of natural origin. Because Zn 5-CQA was synthesized in the solid state therefore its structure in solid state should be carefully described. To this aim the elemental, thermogravimetric and FT-IR analysis were applied. The general formula of the complex was Zn(C_16_H_17_O_9_)_2_∙3H_2_O. The information about thermal stability and thermal decomposition pathway of compound in question was acquired through TG/DSC analysis.

**Figure 3:**
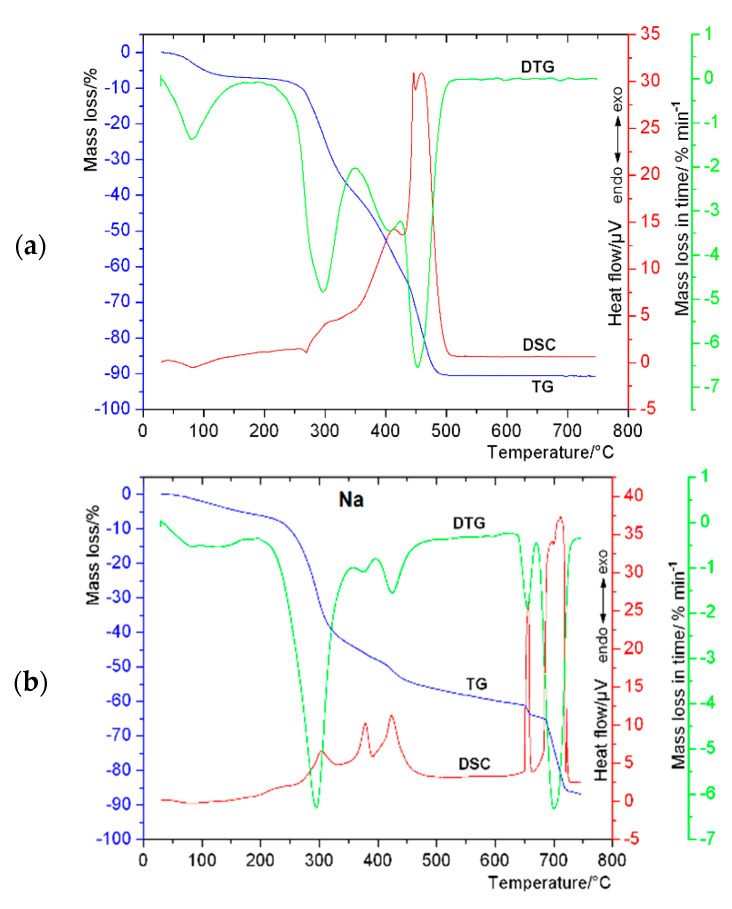
TG-DTG and DSC thermal curves obtained for: (**a**) zinc(II) and (**b**) sodium chlorogenates in air atmosphere.

The analyzed material was stable at room temperature. The thermal decomposition of the title compound proceeded in two main stages related to the dehydration process and gradual degradation of anhydrous material into the metal oxide, which was reflected in the registered thermal profile (Figure 3, Table 4). The release of all water molecules from (Zn(C_16_H_17_O_9_)_2_·3H_2_O) occurred in one evident stage between 30–145 °C which was compliant with the only one observed endothermic effect on the DSC curve with peak top at 83 °C. A mass loss of 6.53% expected for the process of removal of three water molecules was in agreement with the experimentally found value of 6.83%. The anhydrous form (Zn(C_16_H_17_O_9_)_2_) of the studied compound exhibited resistance to thermal decomposition up to about 235 °C. The plateaux of both TG and DSC curves seen between 145 and 235 °C meant fair thermal stability of the anhydrous derivative. Heating above 235 °C lead to the continuous weight loss up to 510 °C. The course of the DSC curve in the temperature range 235–510 °C indicated four energetic effects with the peak maxima located at 269, 413, 446 and 460 °C. First of them was probably related with the endothermic melting process preceding the intense three-step exothermic combustion of the organic part of the complex. The total mass loss of 90.58% (calculated 90.16%) harmonized to the formation of white ZnO as a final product of thermal decomposition. Because Zn(II) chlorogenate was synthesized from Na chlorogenate, the thermal studies for Na salt of chlorogenic acid was performed as well, and the results were shown in Figure 3 and Table 4.

#### 3.3.3. Ultraviolet (UV) Spectra

**Figure 4:**
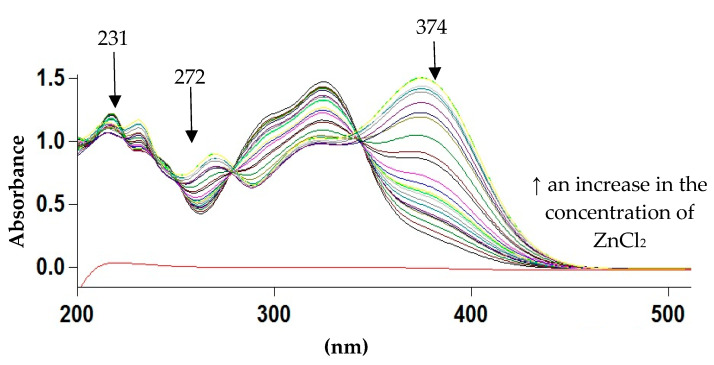
The ultraviolet (UV) spectra of the series of solutions prepared according to the Job’s method (0.1 mM 5-CQA and 0–0.09 mM ZnCl_2_ in Tris-HCl, pH = 7.4). The lowest line showed the spectra of 0.1 mM ZnCl_2_.

**Figure 5:**
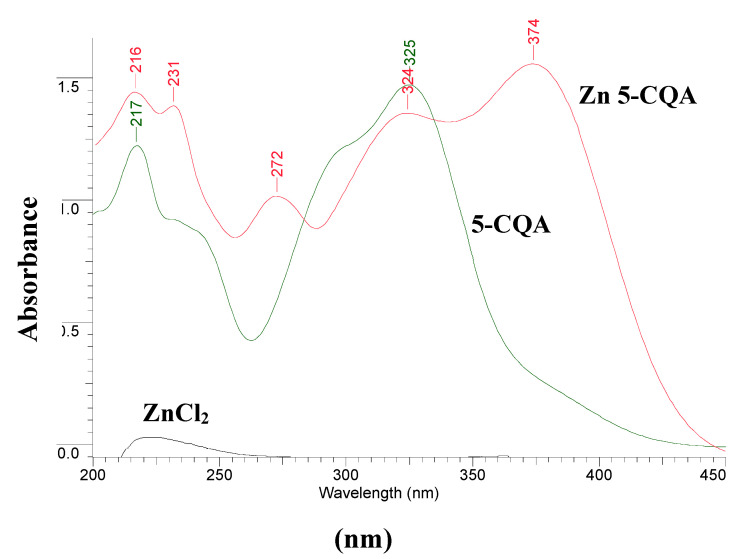
The UV spectra of 0.1 mM ZnCl_2_, 0.1 mM chlorogenic acid (5-CQA), 0.05 mM ZnCl_2_ plus 0.1 mM 5-CQA (molar ratio Zn:5-CQA 1:2) in Tris-HCl at pH = 7.4 registered in the range 200–450 nm.

To study of the composition of Zn 5-CQA in solution the spectrophotometric Job’s method was applied. In the UV spectra of chlorogenic acid four bands at 217, 231, 299 and 325 nm appeared which derived from the π→π* transitions in the frame of aromatic ring and the double bond (Figure 4 and Figure 5) [12]. As a consequence of zinc complex formation the band at 299 nm disappeared, the intensity of the band at 325 nm decreased, and two more bands appeared at 272 and 374 nm. The appearance of the band around 374 nm is characteristic for metal-phenolate interaction and suggests that the catecholate mode was involved in metal ion coordination [30]. With the increase in the concentration of the Zn(II) ions the absorbance of the bands at 231 and 374 increased. The stoichiometry of the complex formed was established by the Job’s method by plotting the absorbance at λ = 374 nm vs. (Zn^2+^)/((Zn^2+^) + (5-CQA)). The maximum of the Job’s curve corresponded to the mole fraction 0.33, confirming the molar ratio Zn:5-CQA 1:2.

In aqueous solution 5-CQA may form various oligomeric structures because of the possibility to bind metal cations through catechol and carboxylate moieties [30]. The other studies showed that Cu(II), Mn(II), Zn(II) and Fe(III) cations formed complexes with chlorogenic acid with the general formula ML_n_ (where L-chlorogenic acid, n = 1, 2 or 3) [29]. The stoichiometry of Cu(II), Fe(II) and Mn(II) chlorogenates at nearly neutral pH was found to be 1:1 [28]. Other studies confirmed that at pH 7.5 the stoichiometry of Cu(II) 5-CQA was 1:1 and 1:2, and for Pb(II) 5-CQA 1:1 [27]. Different coordination of Pb(II) cations by the chlorogenate ligand was shown, i.e., coordination through the carboxylate group in the complex (PbL(H_2_O)_3_)^+^ with stoichiometry 1:1 or with additional binding by the catechol in the complex with stoichiometry 2:1.

### 3.4. Quantum-Chemical Calculations

**Figure 6:**
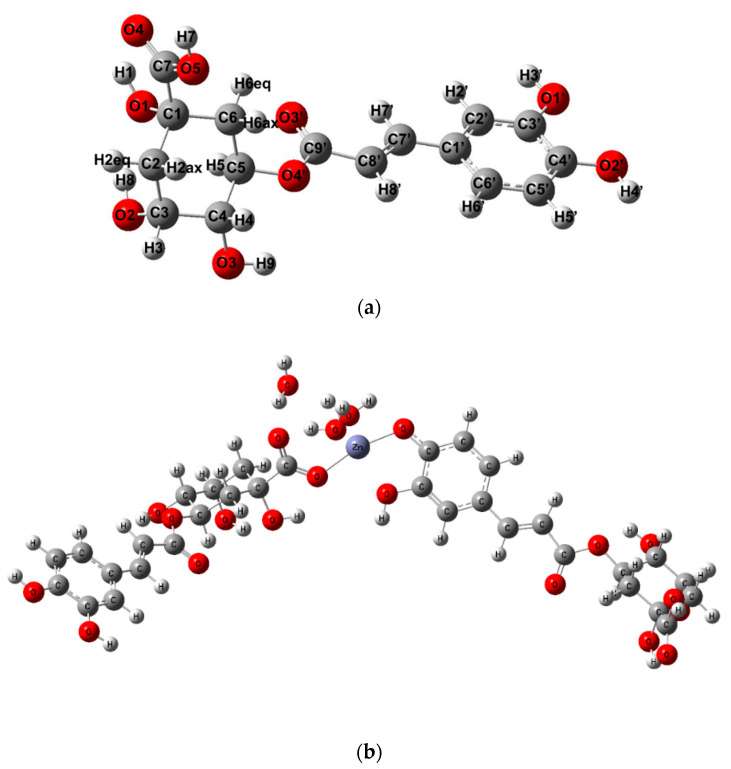

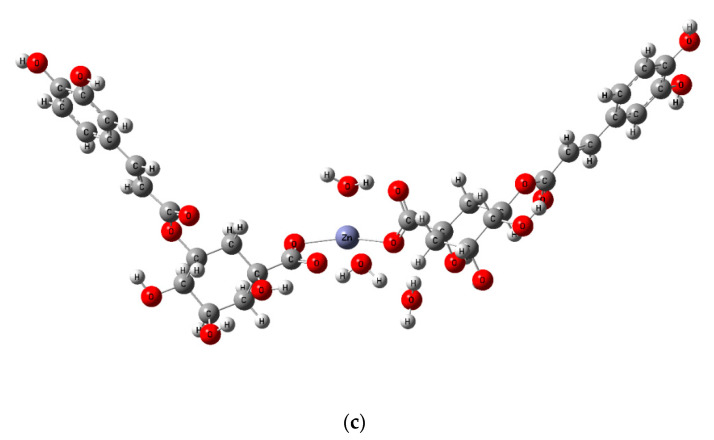
(**a**) The structure of 5-CQA with atom numbering, (**b**) Zn 5-CQA structure I and (**c**) structure II; calculated at B3LYP/6-31G(d) level. The structure I of Zn 5-CQA (b) can be transformed into two different phenoxyl radicals with the participation of: –OH of the catechol group engaged in the Zn^2+^ coordination; –OH from the free catechol group.

The quantum-chemical calculations are complementary to the experimental methods and allow us to predict the structure, atomic charge distribution in molecule, spectral, electronic and thermodynamic parameters which correlate with the biological activity of molecules. The structures of chlorogenic acid and zinc chlorogenate were optimized in the B3LYP/6-31G(d) level (Figure 6). The number of water molecules in a hydrated complex agreed with the results of elemental and thermogravimetric experiments. The two possible ways of metal ion coordination were taken into consideration, i.e., (1) one of the chlorogenate ligand coordinated Zn^2+^ cation via carboxylate group and the second one through the catechol moiety (structure I) or (2) both chlorogenate ligands coordinated the Zn^2+^ cation through the COO^−^ groups (structure II). The selected geometrical parameters (bond lengths and angles) of the studied molecules were gathered in Table S1. The most distinct differences between the structural parameters of ligand and complex concerned the geometry of the –C1-C7O5O4^−^ group of quinic acid moiety engaged in the metal ion coordination. In the structure II of Zn 5-CQA two COO^−^ groups coordinated the Zn^2+^ ion in slightly different manner. They were distinct differences in the C–O lengths and O–C–O angles of particular COO^−^ groups. This should be seen in the experimental FT-IR spectra of complex as a two bands derived from the asymmetric stretching vibrations of the ν_as_(COO^−^) (Figure 2). In the FT-IR spectra of Zn 5-CQA, only one band was assigned to the ν_as_(COO^−^), what may suggest metal ion coordination through only one of the carboxylate anion. In the structure I of Zn 5-CQA, the metal ion was coordinated through the COO^−^ of one ligand and catechol moiety of the second ligand. In such a case the metal ion affected not only the geometry of carboxylate group but also the bond lengths and angles of the skeleton –O1′–C3′–C4′–O2′– in the aromatic ring. These should be seen in the FT-IR spectra of the complex by both (a) the appearance of the bands derived from the ν(COO^−^) vibrations and (b) the shift of the bands assigned to the stretching vibrations of the catechol C–O group (ν (C–O)_catechol group_). In the experimental FT-IR spectra of Zn 5-CQA the band ν (C–O)_catechol group_ was located at much lower wavenumber (1272 cm^−1^) compared with the spectra of acid (1289 cm^−1^). It suggested the participation of catechol group in metal bonding.

The NBO (natural bond orbital) atomic charges calculated for studied molecules were shown in Table S2. The coordination through the carboxylate group slightly increased the positive charge on the C1 and C7 atoms of the quinic acid moiety and increased the negative charge on the O4 and O5 atoms of the carboxylate group. While the coordination via the –OH group of the catechol moiety influenced on the atomic charges of all carbon atoms of the caffeic acid moiety and O3′ and O4′ of the hydroxyl substituents in the ring.

**Figure 7:**
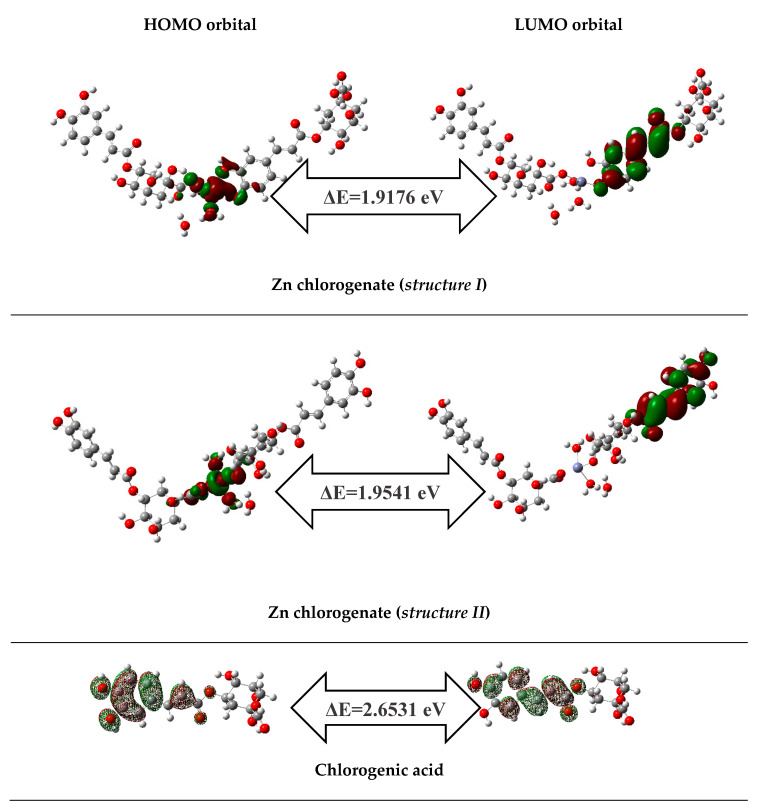
The shapes of frontier molecular orbitals HOMO and LUMO orbitals and energy gaps (ΔE) of chlorogenic acid and Zn chlorogenates calculated at B3LYP/6-31G(d) level.

In Table 5 the electronic parameters calculated on the basis of the theoretical structures of 5-CQA and Zn(II) 5-CQA in the B3LYP/6-31G(d) level were gathered. The antioxidant activity of phenolic compounds is related to the energy of HOMO orbitals which characterizes electron-donating ability of molecule and, therefore, its free radical scavenging efficiency. With the increasing value of HOMO and lowering value of ionic potential (IP), the molecule possesses a rising tendency to donate electrons [52]. The higher HOMO energy and lower ionisation potential of Zn 5-CQA compared to 5-CQA molecule, suggested that the complex had stronger donating electron ability than the ligand. Moreover, Zn 5-CQA possessed a lower energy gap between the LUMO and HOMO orbitals than 5-CQA. This indicated that the complex was more reactive antioxidant than ligand. The type of metal coordination by chlorogenate affected the antioxidant properties of the complex. The energy of HOMO orbital and the energy gap were lower for the Zn 5-CQA where the carboxylate group and catechol moiety both coordinated Zn^2+^, and therefore the structure I of Zn 5-CQA was stronger antioxidant than the structure II (Figure 7). In the case of structure I, the two possibilities can be discussed (Figure 6b) where: (1) * –OH of the catechol group engaged in the Zn^2+^ coordination, or (2) ** –OH from the free catechol group, may be deprotonated in the direct reaction with free radicals. The major antioxidant mechanism is HAT (hydrogen atom transfer) where as a result of a hemolytic O–H bond dissociation the hydrogen atom is transferred from the antioxidant to a free radical. BDE (bond dissociation enthalpy) is a parameter used to estimate the reactivity of the molecule in the HAT mechanism [53]. The lower value of BDE parameter the easier the O–H can be broken. The calculated BDE parameters (Table 6) showed that the abstraction of the hydrogen atom from the –OH group of catechol moiety engaged in the metal ion coordination was much easier than from any other H-atom. It may explain the higher antioxidant activity of Zn 5-CQA revealed in the experiment compared with the ligand alone.

The free radical scavenging properties can be also achieved by a donation of a single electron from the antioxidant to a radical. This mechanism is called a single-electron transfer followed by proton transfer (SET-PT) [44]. It is a two-step mechanism: (I) first the antioxidant reacts with the free radical to form a cation radical of antioxidant and an anion of the radical (ionization potential IP describes the antioxidant reactivity in this mechanism), then (II) the cation radical of the antioxidant decomposes to a radical and a proton (proton dissociation enthalpy PDE parameter describes the reaction). Structure I of the Zn 5-CQA donated the electron most easily (IP = 617.62 kJ/mol) followed by structure II of Zn 5-CQA (IP = 651.71 kJ/mol) and then chlorogenic acid (IP = 707.46 kJ/mol). The PDE parameter which describes the second step of the SET-PT mechanism was much lower for the structure I* of the Zn 5-CQA (the cation radical was formed by the –OH from the catechol moiety that coordinates Zn^2+^) than the other structures of Zn 5-CQA. The lowest value of PDE was for chlorogenic acid what suggested that the 5-CQA contributes to the second step of SET-PT greater than the complexes of 5-CQA.

The third mechanism, a sequential proton-loss electron transfer (SPLET) is realized by a two-step mechanism [44]. First, the antioxidant dissociates into an anion and a proton (proton affinity PA is related with the mechanism). Second, the anion loses an electron and forms the corresponding free radical (electron transfer enthalpy ETE describes the reaction). The first step, i.e., the formation of the phenolate anion, was easier for Zn complexes of chlorogenic acid than for the ligand alone. The lowest PA value was for Zn 5-CQA structure I* which again proved that the antioxidant mechanism with the participation of –OH from the catechol moiety bound to Zn^2+^ is much more favorable than with the unbounded –OH. The second step of the SPLET mechanism is much easier for chlorogenic acid than the Zn(II) complexes.

## 4. Conclusions

Zn(II) cations form stable solution and solid state complexes with chlorogenic acid what cause the change in the pro-/anti-oxidant properties of the ligand. Under exposure to toxic metals, the chelation of Zn(II) by phenolic compounds may effectively increase protection against the oxidative stress in plants, but a higher concentration of formed metal complex may reveal the pro-oxidant activity of phenolic compounds. These may lead to significant damage at the cellular level. Knowledge of the structure of metal-phenolic complexes is important to understand the mechanism of action of phenolic compounds. Moreover, studies on the metal complexes of plant phenolic compounds may contribute to the development of new non-toxic antioxidant or pro-oxidant materials of natural origin which can be used in e.g., plant protection against oxidative stress, food industry or pharmacy.

## Figures and Tables

**Table 1:** Antioxidant properties of the Zn 5-CQA, 5-CQA, L-ascorbic acid, butylated hydroxyanisol (BHA) and butylated hydroxytoluene (BHT) expressed as the ability to scavenge DPPH· radical (EC_50_) and ABTS·^+^ cation radical as well as FRAP and CUPRAC values (the concentrations of tested substances in the samples were * 25 µM, ** 50 µM).

Compound	DPPH/EC_50_ (µM)	ABTS * I%	FRAP ** C_Fe2+_ (µM)	CUPRAC ** C_trolox_ (µM)
Zn 5-CQA	5.45 ± 0.37	97.65 ± 1.25	385.56 ± 5.35	198.36 ± 6.28
5-CQA	7.23 ± 0.76	89.53 ± 3.61	216.09 ± 4.15	129.58 ± 9.70
L-ascorbic acid	10.32 ± 0.98	86.76 ± 1.58	156.86 ± 2.02	25.57 ± 3.50
BHA	13.54 ± 1.61	88.38 ± 2.98	141.82 ± 3.58	83.55 ± 14.97
BHT	53.14 ± 1.05	62.66 ± 5.65	134.40 ± 2.69	74.81 ± 7.57

**Table 2:** The minimum inhibitory concentration (MIC) values (mM) for 5-CQA and Zn 5-CQA against selected microorganisms.

Microorganisms	5-CQA	Zn 5-CQA
*Escherichia coli*	>10	>10
*Pseudomonas aeruginosa*	>10	>10
*Staphylococcus aureus*	>10	1
*Candida albicans*	>10	>10
*Bacillus subtilis*	>10	>10
*Salmonella enteritidis*	>10	>10

**Table 3:** The wavenumbers, intesities and assignment of selected bands from the FT-IR spectra of zinc chlorogenate, sodium chlorogenate [10] and chlorogenic acid [12]; the symbols denote: ν-stretching vibrations, δ-deforming in plane and oop-out of plane bending vibrations; s-strong, m-medium, w-week, v-very, sh-on the slope.

5-CQA [12]		Na 5-CQA [10]		Zn(II) 5-CQA		Assignment
IR	int.	IR	int.	IR	int.	
1725	s	-	-	-	-	v(C=O)_COOH group_
1687	vs	1692	s	1687	s	v(C=O)_ester group_
1640	s	1634	s	1637	sh	v(C=C)
-	-	1598	vs	1616	vs	v(COO^-^)_asym_
1530	m	1528	s	1518	w	v(CC)_ar_
1517	m	-	-	-	-	v(CC)_ar_
1443	s	1450	m	1447	m	δ(COH)_quinic ring_
-	-	1390	sh	1384	m	v(COO^-^)_sym_
1322	m	-		-	-	v(CC) + δ(CCH) + δ(COH)_quinic ring_
1304	s	1323	s	-	-	v(CO) + v(CC) + δ(CCH)
1289	vs	1282	vs	1272	vs	v(C-O)_catechol group_ + δ(CH)_ar_
1251	s	-	-	-	-	v(CC) + v(CH) + δ(CCH) + δ(COH)
1202	s	-	-	-	-	δ(COH)_quinic ring_
1190	vs	1178	vs	1185	m	v(CC) + v(CH) + δ(CCH) + δ(COH)
1159	s	1163	s	1157	m	v(CC) + v(CH) + δ(CCH) + δ(COH)
1134	s	-	-	-	-	v(C-O)_COOH group_
1115	s	1119	s	1123	m	v(C-O)_ester group_
1086	s	1081	s	1083	m	v(CC) + v(C-O)_quinic ring_
1059	w	1059	w	1055	m	v(CC)_quinic ring_ + δ(CH _quinic ring_
1038	m	1037	m	-	-	v(C_quinic ring_-O_in ester group)_ + δ(CH)_quinic ring_
1000	w	997	m	-	-	oop(HC-C=C) + oop(HC=CH)
970	m	969	w	976	w	δ(CH)_quinic ring_ + δ(CH)_quinic ring_ + v(C-O)_quinic ring_
909	m	926	w	914	w	v(CC) + v(C-O)_quinic ring_ + δ(CC) + δ(CH)
853	m	854	w	851	w	δ(HC-CO) + oop(CH)_ester group_
819	s	-	-	-	-	δ(CC)_arom. ring_
-	-	808	m	814	m	δ(CC)_arom. ring_ + δ(COO^−^)
-	-	615	m	617	m	oop(COO^−^)

**Table 4:** Results of thermal decomposition of Zn 5-CQA and Na 5-CQA in air atmosphere. *-endo effect conerns only Zn complex.

Complex	T_1_/°C	T_endo_	Mass Loss/%		Anhydrous Form	T_2_/°C	T_exo_/T_endo_ *	Residue/%		Residue
			Found	Calculated				Found	Calculated	-
Zn(C_16_H_17_O_9_)_2_·3H_2_O	30–145	83	6.83	6.53	Zn(C_16_H_17_O_9_)_2_	235–510	269 * 413 446 460	9.42	9.84	ZnO
Na(C_16_H_17_O_9_)·1.5H_2_O	30–195	79	5.91	6.69	Na(C_16_H_17_O_9_)	195–745	304 379 424 656 713 723	13.35	13.14	Na_2_CO_3_

**Table 5:** Calculated in B3LYP/6-31G(d) level the electronic parameters for 5-CQA and two structures of Zn(II) 5-CQA presented in Figure 6.

Electronic Parameters	5-CQA	Zn(II) 5-CQA (Structure I)	Zn(II) 5-CQA (Structure II)
HOMO (a.u.)	–0.3012	–0.2752	–0.2757
LUMO (a.u.)	–0.2037	–0.2047	–0.2039
HOMO (eV)	–8.1961	–7.4886	–7.5022
LUMO (eV)	–5.5430	–5.5710	–5.5481
GAP (eV)	2.6531	1.9176	1.9541
Ionisation potential (eV)	8.1961	7.4886	7.5022
Affinity (eV)	5.5430	5.5710	5.5481
Global hardness (eV)	1.3266	0.9588	0.9770
Chemical softness (eV)	0.6633	0.4794	0.4885
Electronegativity (eV)	6.8695	6.5298	6.5252
Chemical potential (eV)	–6.8695	–6.5298	–6.5252
Elecrophilicity index (eV)	17.7868	22.2353	21.7894
Total energy (a.u.)	–1297.531	–4602.4041	–4602.4101
Dipole moment (Debay)	5.9017	14.3747	8.8169

**Table 6:** The bond dissociation enthalpies (BDE) (kJ/mol), ionisation potentials (IP) (kJ/mol), proton dissociation enthalpies (PDE) (kJ/mol), proton affinities (PA) (kJ/mol), electron transfer enthalpies (ETE) (kJ/mol) calculated at in B3LYP/6-31G(d) level; Two means of –OH group deprotonation in the direct reaction with free radical are taken into account: * –OH of the catechol group engaged in the Zn^2+^ coordination, or ** –OH from the free catechol group.

Parameter *	5-CQA	Zn(II) 5-CQA (Structure I *)	Zn(II) 5-CQA (Structure I **)	Zn(II) 5-CQA (Structure II)
**BDE**	307.70	224.49	319.72	308.58
**IP**	707.46	617.62	617.62	651.71
**PDE**	909.93	916.58	1011.80	972.76
**PA**	1433.96	1266.58	1385.32	1375.23
**ETE**	189.63	273.81	250.30	249.24

## Supplementary Materials

The following are available online at https://www.mdpi.com/1996-1944/13/17/3745/s1: Table S1: The geometrical parameters for 5-CQA and Zn 5-CQA complexes calculated at B3LYP/6-31G(d) level; atom numbering in Figure 6, Table S2: The NBO atomic charges calculated for 5-CQA and Zn(II) 5-CQA complexes in B3LYP/6-31(d) level; atom numbering in Figure 6.
