# Supplementary material for: Zn(II) Complex of Plant Phenolic Chlorogenic Acid: Antioxidant, Antimicrobial and Structural Studies
Source: Materials (Basel). 2020 Aug 24;13(17):3745. doi: 10.3390/ma13173745 (PMC7504324; doi:10.3390/ma13173745)
Supplement: Supplementary file 1 [file materials-13-03745-s001.pdf]

Table S1. The geometrical parameters for 5-CQA and Zn 5-CQA complexes calculated at B3LYP/6-31G(d) level; atom numbering in Figure 6.

Parameter	5-CQA	Zn 5-CQA (Structure I) *	Zn 5-CQA (Structure II) **
Bond lenghts	-	-	-
C1-C2	1.552	1.553/1.551	1.548/1.546
C2-C3	1.542	1.541/1.542	1.541/1.541
C3-C4	1.542	1.542/1.542	1.542/1.542
C4-C5	1.529	1.529/1.529	1.528/1.528
C5-C6	1.532	1.531/1.533	1.5311.531
C1-C6	1.544	1.541/1.544	1.546/1.540
C1-C7	1.528	1.536/1.527	1.526/1.546
C5-O4′	1.450	1.452/1.448	1.452/1.449
C9′-O4′	1.367	1.366/1.370	1.365/1.366
C9′-O3′	1.216	1.217/1.217	1.217/1.218
C8′-C9′	1.472	1.473/1.468	1.473/1.470
C7′-C8′	1.348	1.348/1.351	1.348/1.349
C1′-C7′	1.458	1.458/1.352	1.458/1.457
C1′-C2′	1.410	1.410/1.414	1.410/1.411
C2′-C3′	1.389	1.389/1.377	1.389/1.389
C3′-C4′	1.410	1.410/1.418	1.410/1.411
C4′-C5′	1.397	1.397/1.410	1.397/1.397
C5′-C6′	1.391	1.391/1.388	1.391/1.391
C1′-C6′	1.404	1.404/1.410	1.404/1.404
C3′-O1′	1.364	1.364/1.396	1.365/1.364
C4′-O2′	1.361	1.361/1.319	1.361/1.360
C1-O1	1.430	1.433/1.430	1.432/1.445
C3-O2	1.415	1.416/1.415	1.416/1.418
C4-O3	1.413	1.413/1.413	1.413/1.413
O2′-Zn^{2+}	-	-/1.923	-
O4/O5-Zn^{2+}	-	1.996/-<br>3.138/-	2.216/3.205<br>2.075/1.987
O4/O5-C7	1.215<br>1.344	1.263/1.215<br>1.278/1.344	1.279/1.284<br>1.262/1.248
H1-O4	2.011	1.970/2.012	1.962/2.881
O1′-Zn^{2+}	-	-/2.161	-
Angles	-	-	-
O4-C7-O5	123.385	125.110/123.324	120.840/125.701
O1′-C3′-C4′	116.979	116.975/112.79	116.997/116.946
C3′-C4′-O2′	117.209	117.201/120.186	117.216/117.174
O1′-C3′-C4′-O2′	−0.053	0.012/0.419	−0.010/−0.009

* structure I–metal cation is coordinated via the COO^- group and –OH group from the catechol moiety; in the case of structure I the first column present the data for ligand linked to metal ion via the COO^- group, and the second one show the data for ligand bonded to metal ion by the catechol part. ** structure II–metal cation is coordinated by two COO^- groups from the quinic moiety

Table S2. The NBO atomic charges calculated for 5-CQA and Zn(II) 5-CQA complexes in B3LYP/6-31(d) level; atom numbering in Figure 6.

Atom	5-CQA	Zn(II) 5-CQA (Structure I) *	Zn(II) 5-CQA (Structure II) **
C1	0.186	0.1948/0.186	0.197/0.197
C2	−0.509	−0.509/−0.509	−0.506/−0.507
C3	0.082	0.082/0.082	0.082/0.081

C4	0.060	0.060/0.060	0.060/0.061
C5	0.051	0.051/0.051	0.050/0.051
C6	−0.503	−0.501/−0.502	−0.499/−0.505
C7	0.835	0.855/0.835	0.803/0.846
C1′	−0.102	−0.102/−0.120	−0.102/−0.103
C2′	−0.268	−0.269/−0.259	−0.269/−0.268
C3′	0.285	0.285/0.232	0.285/0.285
C4′	0.301	0.301/0.339	0.301/0.302
C5′	−0.301	−0.301/−0.287	−0.301/−0.301
C6′	−0.205	−0.205/−0.195	−0.205/−0.204
C7′	−0.136	−0.138/−0.135	−0.138/−0.134
C8′	−0.344	−0.342/−0.358	−0.342/−0.344
C9′	0.790	0.790/0.787	0.790/0.792
O1	−0.781	−0.782/−0.780	−0.790/−0.775
O2	−0.763	−0.764/−0.763	−0.765/−0.761
O3	−0.742	−0.742/−0.743	−0.743/−0.742
O4	−0.609	−0.759/−0.610	−0.725/−0.700
O5	−0.697	−0.770/−0.697	−0.755/−0.799
O1′	−0.674	−0.675/−0.737	−0.675/−0.674
O2′	−0.666	−0.667/−0.813	−0.667/−0.665
O3′	−0.608	−0.609/−0.614	−0.610/−0.613
O4′	−0.574	−0.574/−0.577	−0.573/−0.570

* structure I–metal cation is coordinated via the COO^- group and –OH group from the catechol moiety; in the case of structure I the first column present the data for ligand linked to metal ion via the COO^- group, and the second one show the data for ligand bonded to metal ion by the catechol part ** structure II–metal cation is coordinated by two COO^- groups from the quinic moiety.

© 2020 by the authors. Submitted for possible open access publication under the terms and conditions of the Creative Commons Attribution (CC BY) license (http://creativecommons.org/licenses/by/4.0/).
